# A nomogram to predict the risk of venous thromboembolism in patients with colon cancer in China

**DOI:** 10.1002/cam4.7231

**Published:** 2024-05-02

**Authors:** Yuanyuan Yang, Jiayi Zhan, Xiaosheng Li, Jun Hua, Haike Lei, Xiaoliang Chen

**Affiliations:** ^1^ Department of Nuclear Medicine Chongqing University Cancer Hospital Chongqing China; ^2^ Department of Traditional Chinese Medicine Chongqing University Cancer Hospital Chongqing China; ^3^ Chongqing Cancer Multi‐omics Big Data Application Engineering Research Center Chongqing University Cancer Hospital Chongqing China

**Keywords:** colon cancer, nomogram, predictive model, venous thromboembolism

## Abstract

**Objective:**

To create a nomogram for predicting the likelihood of venous thromboembolism (VTE) in colon cancer patients from China.

**Methods:**

The data of colon cancer patients from Chongqing University Cancer Hospital between 2019 and 2022 were analyzed. Patients were divided into training set and internal validation set by random split‐sample method in a split ratio of 7:3. The univariable and multivariable logistic analysis gradually identified the independent risk factors for VTE. A nomogram was created using all the variables that had a significance level of *p* < 0.05 in the multivariable logistic analysis and those with clinical significance. Calibration curves and clinical decision curve analysis (DCA) were used to assess model's fitting performance and clinical value. Harrell's C‐index (concordance statistic) and the area under the receiver operating characteristic curves (AUC) were used to evaluate the predictive effectiveness of models.

**Results:**

A total of 1996 patients were ultimately included. There were 1398 patients in the training set and 598 patients in the internal validation set. The nomogram included age, chemotherapy, targeted therapy, hypertension, activated partial thromboplastin time, prothrombin time, platelet, absolute lymphocyte count, and D‐dimer. The C‐index of nomogram and Khorana score were 0.754 (95% CI 0.711–0.798), 0.520 (95% CI 0.477–0.563) in the training cohort and 0.713 (95% CI 0.643–0.784), 0.542 (95% CI 0.473–0.612) in the internal validation cohort.

**Conclusions:**

We have established and validated a nomogram to predict the VTE risk of colon cancer patients in China, which encompasses a diverse age range, a significant population size, and various clinical factors. It facilitates the identification of high‐risk groups and may enable the implementation of targeted preventive measures.

## 1 INTRODUCTION

Colon cancer, characterized by a high incidence and mortality rate, stands as one of the most prevalent diseases worldwide. During the sole year of 2020, the world witnessed more than 1.9 million new instances of colorectal cancer (including the anus), resulting in an estimated 935,000 fatalities spread across 185 nations. Approximately one out of every 10 cancer cases and deaths can be attributed to this astonishing statistic.^1^, ^2^ Moreover, colorectal cancer ranks among the top five diagnosed cancers and represents a leading cause of tumor‐related mortality in China.^3^, ^4^ In cancer patients, the occurrence of venous thromboembolism (VTE), which includes deep vein thrombosis (DVT) and/or pulmonary embolism (PE), is a serious complication that leads to increased mortality and reduced quality of life.^5^, ^6^ People who have been diagnosed with cancer have a risk of developing VTE that is six to seven times higher than those without cancer.^7^ The occurrence of VTE elevates the 6‐month mortality risk for tumor patients, with estimates ranging from 29% to 94%, marking a threefold increase.^8^ This underscores the critical need for an in‐depth exploration of factors linked to a high risk of VTE, particularly in tumor types predisposed to this complication, such as gastrointestinal cancer—ranking third in frequency after hematologic cancer and lung cancer.^9^

Numerous studies have investigated the factors that increase the likelihood of VTE in patients with colorectal cancer in Western countries as well as several Asian nations. A study^10^ was carried out in America to investigate the occurrence of VTE and the rates of death in the elderly (≥65 years old) diagnosed with Stage III colon cancer, where 82% patients were non‐Hispanic White. Similarly, in Japan, risk factors for VTE were identified in patients who were at least 65 years old and had advanced diseases, including the presence of distant metastases and an ECOG PS score of 2.^11^ M. M. I. Vendler et al.^12^ and Song‐Soo Yang et al.^13^ separately highlighted VTE risk for patients received colorectal cancer surgery in Denmark and Korea. Unfortunately, the understanding in China regarding the risk of VTE in patients with colon cancer is lack, and there is a scarcity of data on developing a VTE prediction model specifically for colon cancer patients to assist in clinical decision‐making.

Khorana score (KS) has gained significant popularity in clinical practice to predict the incidence of VTE in patients with cancer initiating chemotherapy.^14^, ^15^, ^16^ Nevertheless, KS has certain limitations and is suitable for specific subgroups. KS is more commonly applied in outpatient settings excluding most hematologic malignancy and modern noncytotoxic treatments.^16^, ^17^ Colon cancer is a separate subgroup with distinct features like genetic mutations and various treatment choices. And the majority of colon cancer patients underwent surgery when they were available. KS might not be the top priority for establishing a predictive model for VTE probability in colon cancer patients, as it may not cover all relevant risk factors specific to this group such as VTE occurring during hospitalization. The nomogram, serving as both an integrated model and a tool for personalized medicine, presents a compelling alternative.^18^ Furthermore, it converts the traditional regression model into a visually intuitive graphical evaluation for individual, providing physicians with beneficial practicality and accuracy. There are ample evidence confirms the effectiveness of the nomogram in accurately evaluating the risk of VTE in different types of cancer, such as lung cancer,^17^ breast cancer,^19^ ovarian cancer,^20^ and other malignancies. The nomogram model has been effectively used to forecast the risk of VTE in individuals with lymphoma, exhibiting a significant agreement between projected and real probabilities of VTE, surpassing the performance of the Khorana model.^21^

Hence, the primary objective of this study is to develop a nomogram model specifically tailored to the Chinese population with colon cancer to provide a precise and reliable prediction of the probability of VTE risk. Our study employs a robust methodology, drawing on a substantial sample size and encompassing a comprehensive array of risk factors to enhance the accuracy and applicability of nomogram.

## 2 MATERIALS AND METHODS

### 2.1 Patient population

From 2019 to 2022, participants for this study were recruited from Chongqing University Cancer Hospital in China. Patients who had at least once hospitalization with a confirmed diagnosis of colon cancer were included. Individuals who were under 18 years old, had a previous occurrence of VTE before being diagnosed with colon cancer, died within 48 h after admission, or had incomplete medical records were excluded. To optimize the robustness and generalizability of the findings, all available data in the database were utilized. Notably, for patients with multiple hospital stays, their data were included only once in the analysis. This study was conducted under license in accordance with the guidelines outlined in the Declaration of Helsinki and applicable local regulations. Approval for the study was obtained from the Ethics Committee of Chongqing University Cancer Hospital (CZLS2023337‐A). Informed consent was waived given the retrospective nature of the study.

### 2.2 Diagnosis of VTE

We defined VTE as DVT, PE, and superficial thrombophlebitis. The diagnosis of DVT was mainly based on the findings of ultrasound imaging, including intravascular thrombosis, and interruption of blood flow signal. Patients who suffered from redness, swelling, and pain in the skin along the vascular path without intravascular thrombosis were diagnosed with superficial thrombophlebitis. PE was diagnosed primarily through single/bilateral/multilobar pulmonary artery embolism in CTPA with/without typical clinical symptoms like dyspnea, chest pain, and hemoptysis.^22^, ^23^, ^24^ Two experienced radiologists identified and reviewed the above diagnosis independently. If their conclusions were inconsistent, a third radiologist identified the diagnosis, and his/her diagnosis was regarded as the final result.

### 2.3 Construction and calibration of the nomogram model

Patients were screened according to the inclusion and exclusion criteria. Those included were randomly divided into a training set and an internal validation set^25^ at a ratio of 7:3 with blind to their information including age, Karnofsky Performance Status (KPS), body mass index (BMI), gender, with or without VTE. Feature selection was meticulously conducted on the training set, striking a balance between model performance and clinical applicability to formulate an effective clinical prediction model. Initially, the association between VTE and clinical variables was scrutinized through univariable logistic regression analysis. Afterwards, variables showing significance with a *p* value less than 0.05 in the univariable analysis and those considered clinically significant, were included in the multivariable logistic analysis. The variables identified as effective predictors of VTE in the multivariable analysis were then utilized to construct the nomogram model. The nomogram was internally validated. The “NomogramEx” package in R was utilized to calculate scores for each variable, enabling the determination of total scores for each patient. Calibrating ability was evaluated by using calibration plots.

### 2.4 Comparison of the nomogram model with KS

The discrimination of the predictive model was assessed using Harrell's C‐index (concordance statistic) and the area under the receiver operating characteristic curves (AUC) in both training and internal validation sets. Furthermore, the utilization of the “rmda” software package was applied to perform decision curve analysis (DCA) to assess the practicality of the model in a clinical setting. The abscissa of this graph is the threshold probability. It reflects a continuum of VTE risk thresholds where the occurrence of VTE for patient i was recorded as Pi. When Pi reaches a certain threshold (denoted as Pt), it is defined as positive which illustrates some interventions (such as changing the anticoagulation regimen) should be taken into consideration. The two references represent two extreme cases. The horizontal one indicates that all samples are negative, with no intervention for all and no net benefit. While the slanted one means that all samples received intervention. The net benefit is a backslope with a negative slope. Comparing the C‐index and net benefit of nomogram model with KS to evaluate their predictive ability and performance.

### 2.5 Statistical analysis

Descriptive statistics for continuous variables that follow a normal distribution were reported as the arithmetic mean plus or minus the standard deviation (mean ± SD). The *t*‐test was used to evaluate the statistical significance of differences between two groups. When the data deviated from a normal distribution, the median (M) and interquartile range (IQR) were used for presentation, and nonparametric tests were used to compare the differences between groups. Count data were expressed as frequencies and percentages, and comparisons were conducted using chi‐squared tests. In nomogram model, continuous variables were transformed into categorical variables (low risk or high risk) by determining the optimal cut‐off value according to the maximum Youden index on the basis of receiver operating characteristic (ROC) curves. Five variables were identified in the Khorana model in which the patients whose score equal to or greater than 3 were considered high‐risk group.^15^ R software version 4.1.2 (http//www.r‐project.org) was utilized for conducting all statistical analyses, with a predetermined significance level of 0.05 for all outcomes.

## 3 RESULTS

### 3.1 Demographics and clinicopathologic characteristics of all patients

**FIGURE 1:**
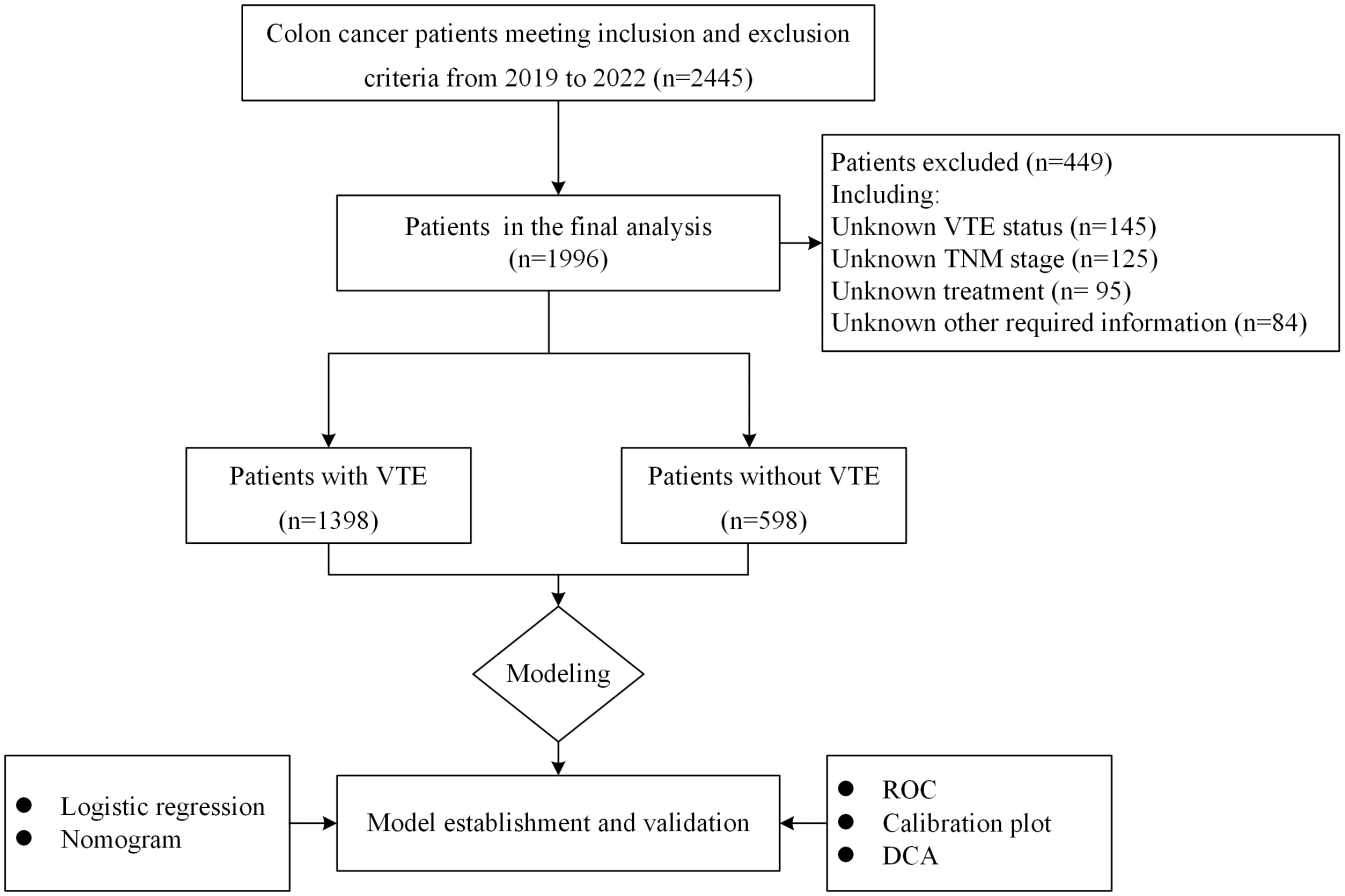
Flow chart of the patients enrolled in the final study cohorts.

From 2019 to 2022, our study screened a total of 2445 individuals diagnosed with colon cancer. Out of these, 1996 patients were eventually enrolled (Figure 1). Among them, 182 patients were diagnosed with VTE, constituting 9.1% of the total study population. Eighteen variables were recorded for each patient. The age of the individuals was 60.33 ± 12.63 years, with males accounting for 42.48% (848 out of 1996). The majority of patients (69.94%, 1396 out of 1996) presented with Stage III/IV. Significant variations were observed in age, BMI, TNM stage, radiation, chemotherapy, targeted therapy, hypertension, prothrombin time (PT), absolute lymphocyte count (LYM), platelet count (PLT), and D‐dimer distribution between the two groups, VTE or non‐VTE (*p* < 0.05). A summary of the demographics and clinicopathologic characteristics of the patients is provided in Table 1.

**TABLE 1:** Demographics and clinicopathologic characteristics of colon cancer patients with or without VTE.

Variables	Overall (*n* = 1996)	Non‐VTE (*n* = 1814)	VTE (*n* = 182)	*p*
Age (year) (mean ± SD)	60.33 ± 12.63	60.00 ± 12.65	63.54 ± 12.07	<0.001
KPS (points) (mean ± SD)	81.71 ± 8.53	81.80 ± 8.44	80.85 ± 9.31	0.153
BMI (kg/m^2^) (*n* [%])				
18.5–23.9	120 (6.01)	100 (5.51)	20 (10.99)	0.027
24–27.9	1111 (55.66)	1015 (55.95)	96 (52.75)	
≥28	625 (31.31)	573 (31.59)	52 (28.57)	
<18.5	140 (7.01)	126 (6.95)	14 (7.69)	
Sex (*n* [%])				
Female	1148 (57.52)	1042 (57.44)	106 (58.24)	0.897
Male	848 (42.48)	772 (42.56)	76 (41.76)	
Histology (*n* [%])				
Adenocarcinoma	1980 (99.20)	1800 (99.23)	180 (98.90)	0.971
Others	16 (0.80)	14 (0.77)	2 (1.10)	
TNM (*n* [%])				
I–II	600 (30.06)	560 (30.87)	40 (21.98)	0.005
III	542 (27.15)	498 (27.45)	44 (24.18)	
IV	854 (42.79)	756 (41.68)	98 (53.85)	
Radiation (*n* [%])				
No	1841 (92.23)	1681 (92.67)	160 (87.91)	0.032
Yes	155 (7.77)	133 (7.33)	22 (12.09)	
Chemotherapy (*n* [%])				
No	840 (42.08)	783 (43.16)	57 (31.32)	0.003
Yes	1156 (57.92)	1031 (56.84)	125 (68.68)	
Surgery (*n* [%])				
No	750 (37.58)	683 (37.65)	67 (36.81)	0.887
Yes	1246 (62.42)	1131 (62.35)	115 (63.19)	
Immunotherapy (*n* [%])				
No	1955 (97.95)	1779 (98.07)	176 (96.70)	0.334
Yes	41 (2.05)	35 (1.93)	6 (3.30)	
Targeted therapy (*n* [%])				
No	1588 (79.56)	1482 (81.70)	106 (58.24)	<0.001
Yes	408 (20.44)	332 (18.30)	76 (41.76)	
Hypertension (*n* [%])				
No	1533 (76.80)	1418 (78.17)	115 (63.19)	<0.001
Yes	463 (23.20)	396 (21.83)	67 (36.81)	
Diabetes (*n* [%])				
No	1734 (86.87)	1581 (87.16)	153 (84.07)	0.288
Yes	262 (13.13)	233 (12.84)	29 (15.93)	
APTT (s) (mean ± SD)	27.60 ± 3.85	27.62 ± 3.75	27.33 ± 4.80	0.334
PT (s) (mean ± SD)	11.82 ± 1.35	11.79 ± 1.31	12.11 ± 1.73	0.003
LYM (10^9^/L) (mean ± SD)	1.42 ± 0.55	1.44 ± 0.54	1.24 ± 0.61	<0.001
PLT (10^9^/L) (mean ± SD)	249.17 ± 109.10	251.81 ± 109.99	222.93 ± 96.13	<0.001
D‐dimer (mg/L) (*n* [%])				
≤0.5	740 (37.07)	708 (39.03)	32 (17.58)	<0.001
>0.5	1256 (62.93)	1106 (60.97)	150 (82.42)	

Abbreviations: APTT, activated partial thromboplastin time; LYM, absolute lymphocyte count; PLT, platelet; PT, prothrombin time.

### 3.2 Characteristics of the training and validation sets

Using the random split‐sample method, 1398 out of 1996 patients were assigned to the training cohort, while the remaining 598 patients were allocated to the internal validation set, maintaining a split ratio of 7:3. There were no notable disparities observed between the two groups, as outlined in Table 2.

**TABLE 2:** Clinical characteristics of the training and validation sets.

Variables	Overall (*n* = 1996)	Training set (*n* = 1398)	Validation set (*n* = 598)	*p*
Age (year) (mean ± SD)	60.33 ± 12.63	60.01 ± 12.57	61.07 ± 12.76	0.085
KPS (points) (mean ± SD)	81.71 ± 8.53	81.73 ± 8.55	81.67 ± 8.48	0.881
BMI (kg/m^2^) (*n* [%])				
18.5–23.9	120 (6.01)	83 (5.94)	37 (6.19)	0.628
24–27.9	1111 (55.66)	781 (55.87)	330 (55.18)	
≥28	625 (31.31)	430 (30.76)	195 (32.61)	
<18.5	140 (7.01)	104 (7.44)	36 (6.02)	
Sex (*n* [%])				
Female	1148 (57.52)	810 (57.94)	338 (56.52)	0.591
Male	848 (42.48)	588 (42.06)	260 (43.48)	
Histology (*n* [%])				
Adenocarcinoma	1980 (99.20)	1384 (99.00)	596 (99.67)	0.209
Others	16 (0.80)	14 (1.00)	2 (0.33)	
TNM (*n* [%])				
I–II	600 (30.06)	428 (30.62)	172 (28.76)	0.682
III	542 (27.15)	379 (27.11)	163 (27.26)	
IV	854 (42.79)	591 (42.27)	263 (43.98)	
Radiation (*n* [%])				
No	1841 (92.23)	1297 (92.78)	544 (90.97)	0.197
Yes	155 (7.77)	101 (7.22)	54 (9.03)	
Chemotherapy (*n* [%])				
No	840 (42.08)	585 (41.85)	255 (42.64)	0.779
Yes	1156 (57.92)	813 (58.15)	343 (57.36)	
Surgery (*n* [%])				
No	750 (37.58)	531 (37.98)	219 (36.62)	0.600
Yes	1246 (62.42)	867 (62.02)	379 (63.38)	
Immunotherapy (*n* [%])				
No	1955 (97.95)	1374 (98.28)	581 (97.16)	0.146
Yes	41 (2.05)	24 (1.72)	17 (2.84)	
Targeted therapy (*n* [%])				
No	1588 (79.56)	1128 (80.69)	460 (76.92)	0.064
Yes	408 (20.44)	270 (19.31)	138 (23.08)	
Hypertension (*n* [%])				
No	1533 (76.80)	1065 (76.18)	468 (78.26)	0.342
Yes	463 (23.20)	333 (23.82)	130 (21.74)	
Diabetes (*n* [%])				
No	1734 (86.87)	1221 (87.34)	513 (85.79)	0.385
Yes	262 (13.13)	177 (12.66)	85 (14.21)	
APTT (s) (mean ± SD)	27.60 ± 3.85	27.58 ± 3.85	27.64 ± 3.87	0.758
PT (s) (mean ± SD)	11.82 ± 1.35	11.81 ± 1.34	11.85 ± 1.39	0.530
LYM (10^9^/L) (mean ± SD)	1.42 ± 0.55	1.42 ± 0.54	1.42 ± 0.57	0.865
PLT (10^9^/L) (mean ± SD)	249.17 ± 109.10	248.02 ± 109.08	251.88 ± 109.20	0.469
D‐dimer (mg/L) (*n* [%])				
≤0.5	740 (37.07)	530 (37.91)	210 (35.12)	0.257
>0.5	1256 (62.93)	868 (62.09)	388 (64.88)	

Abbreviations: APTT, activated partial thromboplastin time; LYM, absolute lymphocyte count; PLT, platelet; PT, prothrombin time.

### 3.3 Significant predictive factors in the training set

In the training cohort, 131 patients (9.37%) experienced VTE, while none VTE occurred in 1267 patients (90.63%). Table 3 displays the findings from both univariable and multivariable logistic analyses. Significant predictive factors influencing VTE were identified through univariable analysis, including age, chemotherapy, targeted therapy, hypertension, activated partial thromboplastin time (APTT), LYM, PLT, and D‐dimer. And then, the multivariable analysis revealed positive correlations between age, chemotherapy, targeted therapy, hypertension, APTT, PT, PLT, D‐dimer, and VTE. Particularly, patients who had previously undergone chemotherapy (OR = 1.95 [1.23–3.09], *p* = 0.005), targeted therapy (OR = 2.50 [1.63–3.84], *p* < 0.001), or had hypertension (OR = 1.67 [1.11–2.53], *p* = 0.014) exhibited a statistically significant increase in the risk of VTE. Furthermore, individuals with reduced APTT (OR = 0.93 [0.88–0.98], *p* = 0.011) or decreased PLT concentrations (OR = 1.00 [1.00–1.00], *p* = 0.018) exhibited an elevated susceptibility to VTE in contrast to those with higher levels. D‐dimer level over 0.5 were linked to an increased risk of VTE (OR = 3.09 [1.87–5.09], *p* < 0.001). Surprisingly, the multivariable logistic analysis did not prove any statistically significant distinction between LYM and VTE, which contradicted the outcome observed in the univariable analysis.

**TABLE 3:** Logistic regression analysis of the risk factors for VTE in the training set.

Variables	OR (univariable)	OR (multivariate)
Age (year)	1.03 (1.01–1.04, *p* < 0.001)	1.03 (1.01–1.04, *p* = 0.005)
KPS (points)	0.99 (0.97–1.01, *p* = 0.441)	
Sex		
Female		
Male	0.81 (0.56–1.17, *p* = 0.258)	
Histology		
Adenocarcinoma		
Others	0.74 (0.10–5.72, *p* = 0.775)	
TNM		
I–II		
III	1.22 (0.73–2.05, *p* = 0.446)	
IV	1.75 (1.12–2.74, *p* = 0.014)	
Chemotherapy		
No		
Yes	1.94 (1.30–2.88, *p* = 0.001)	1.95 (1.23–3.09, *p* = 0.005)
Surgery		
No		
Yes	0.99 (0.68–1.44, *p* = 0.963)	
Immunotherapy		
No		
Yes	1.96 (0.66–5.83, *p* = 0.224)	
Targeted therapy		
No		
Yes	3.05 (2.09–4.46, *p* < 0.001)	2.50 (1.63–3.84, *p* < 0.001)
Hypertension		
No		
Yes	2.07 (1.42–3.02, *p* < 0.001)	1.67 (1.11–2.53, *p* = 0.014)
Diabetes		
No		
Yes	1.45 (0.89–2.36, *p* = 0.137)	
APTT (s)	0.95 (0.90–0.99, *p* = 0.028)	0.93 (0.88–0.98, *p* = 0.011)
PT (s)	1.06 (0.94–1.21, *p* = 0.324)	1.18 (1.01–1.38, *p* = 0.033)
LYM (10^9^/L)	0.54 (0.37–0.79, *p* = 0.001)	0.70 (0.47–1.02, *p* = 0.066)
PLT (10^9^/L)	1.00 (1.00–1.00, *p* = 0.005)	1.00 (1.00–1.00, *p* = 0.018)
D‐dimer (mg/L)		
≤0.5		
>0.5	3.52 (2.18–5.68, *p* < 0.001)	3.09 (1.87–5.09, *p* < 0.001)

Abbreviations: APTT, activated partial thromboplastin time; LYM, absolute lymphocyte count; OR, odds ratio; PLT, platelet; PT, prothrombin time.

### 3.4 Construction and calibration of the nomogram model

**FIGURE 2:**
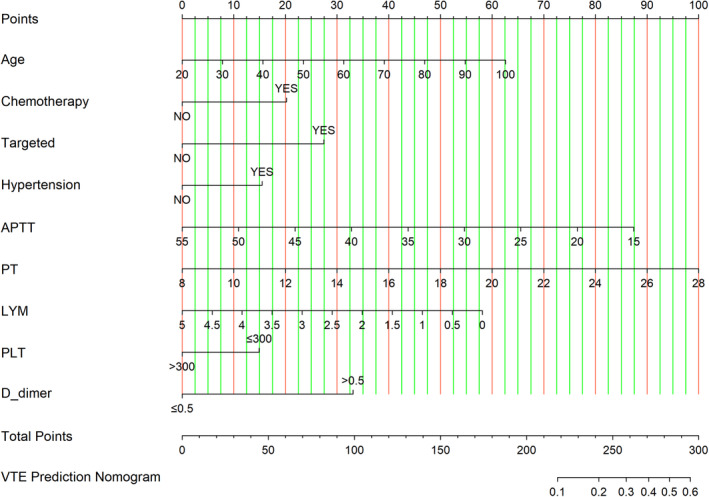
The nomogram for predicting VTE risk of colon cancer patients. VTE, venous thromboembolism.

A nomogram was developed based on the significant predictive factors identified in the multivariable logistic regression analysis, as well as factors with clinical significance (refer to Figure 2). A score on the point scale was assigned to every variable. The total scores were subsequently computed by summing the scores for each factor, and the corresponding estimated likelihood of VTE was ascertained by locating the cumulative scores on the scale. According to the nomogram, PT had the largest impact on the prediction of VTE, followed by APTT, PLT, age, and LYM. Chemotherapy, targeted therapy, high blood pressure, and D‐dimer showed a moderate impact on predicting VTE in individuals diagnosed with colon cancer.

**FIGURE 3:**
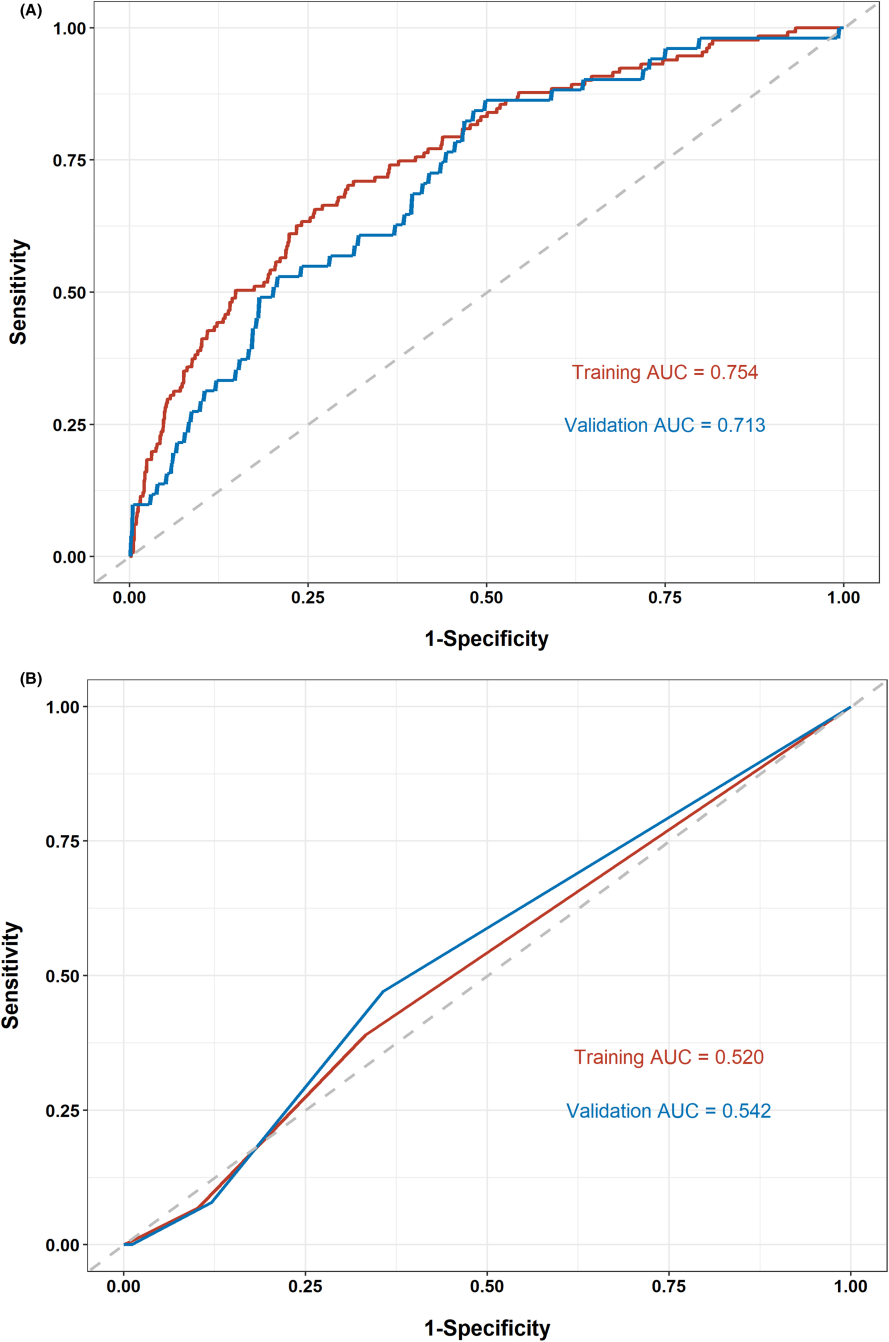
Calibration plots of the nomogram for VTE risk in the training cohort (A) and internal validation cohort (B). VTE, venous thromboembolism.

The calibration curves demonstrated that the concordance between the anticipated and witnessed occurrences of VTE was excellent in training set (Figure 3A) and not bad in the internal validation (Figure 3B).

### 3.5 Comparison of the nomogram model with KS

**FIGURE 4:**
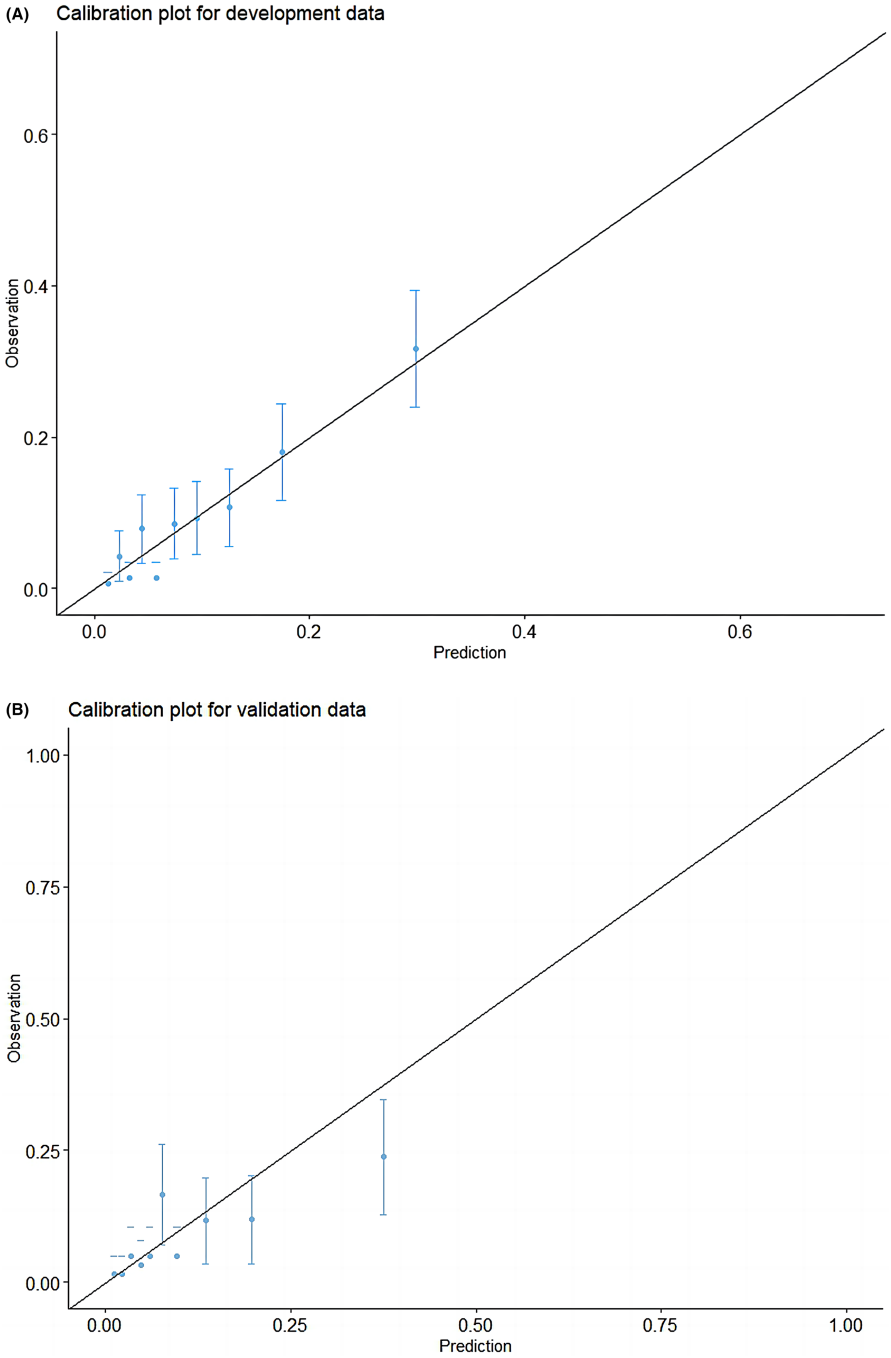
ROC curves of the nomogram (A) and KS (B) for VTE risk prediction in the training and internal validation cohorts. KS, Khorana score; ROC, receiver operating characteristic curve; VTE, venous thromboembolism.

The established nomogram showed a C‐index of 0.754 with a 95% CI of 0.711–0.798 in the training group and 0.713 with a 95% CI of 0.643–0.784 in the internal validation group (Figure 4A). The C‐index of KS was 0.520 (95% CI 0.477–0.563) in the training group and 0.542 (95% CI 0.473–0.612) in the internal validation group (Figure 4B) which were both lower than those of nomogram. As can be seen from Table 4, the diagnosis rate of non‐VTE was 95.82% (872 out of 910) of nomogram and 90.31% (1137 out of 1259) of KS for low‐risk population in the training set and 94.55% (364 out of 385) of nomogram, and 91.10% (481 out of 528) of KS in the internal validation set. As far as high‐risk population are concerned, the diagnosis rate of VTE was 19.06% (93 out of 488) of nomogram and 6.47% (9 out of 139) of KS in the training set and 14.08% (30 out of 213) of nomogram, and 5.71% (4 out of 70) of KS in the internal validation set.

**TABLE 4:** Comparison of the nomogram model with KS.

Variables	Nomogram				KS			
	Training set		Validation set		Training set		Validation set	
	Low risk	High risk	Low risk	High risk	Low risk	High risk	Low risk	High risk
Non‐VTE	872 (95.82)	395 (80.94)	364 (94.55)	183 (85.92)	1137 (90.31)	130 (93.53)	481 (91.1)	66 (94.29)
VTE	38 (4.18)	93 (19.06)	21 (5.45)	30 (14.08)	122 (9.69)	9 (6.47)	47 (8.9)	4 (5.71)

Abbreviation: KS, Khorana score.

**FIGURE 5:**
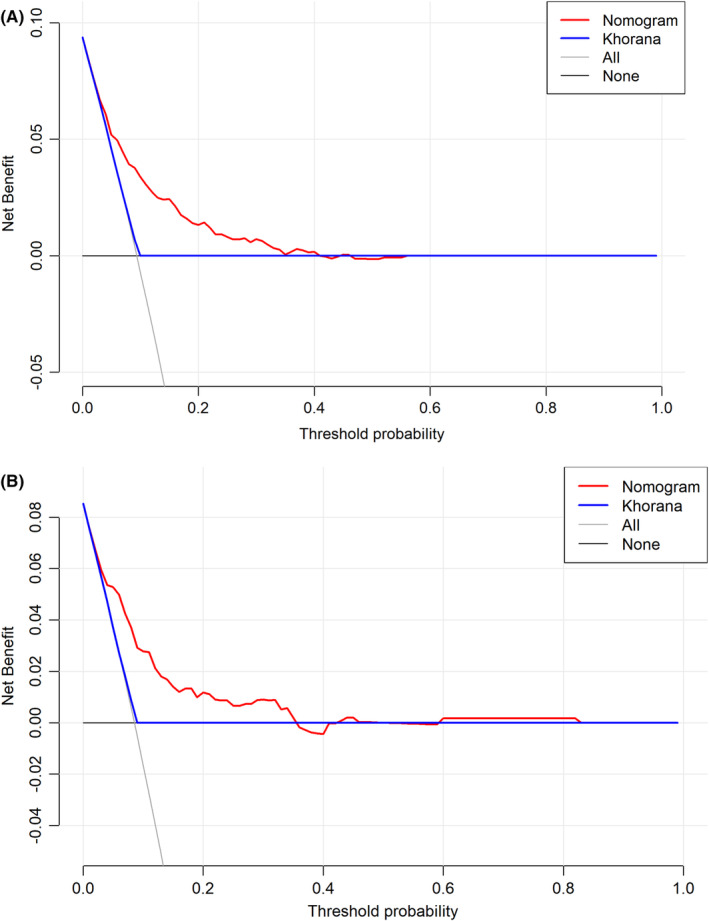
DCA of the nomogram and KS for VTE in the training cohort (A) and internal validation cohort (B). DCA, decision curve analysis; KS, Khorana score; VTE, venous thromboembolism.

The DCA curves of nomogram demonstrated more benefit within a probability range of 5%–40% in the training set (Figure 5A) and 5%–42% in the internal validation set (Figure 5B) than either treating none or treating all patients.

## 4 DISCUSSION

Malignant cancers are often accompanied by VTE, which is the second leading cause of death following the cancer diagnosis itself.^10^ The incidence of VTE varies across different types of cancer, with certain cancers, such as colon cancer, exhibiting a higher VTE incidence. This heightened incidence may be indicative of a more aggressive biological behavior.^26^ Therefore, it is crucial to prioritize the identification and comprehension of the risk elements associated with VTE in these particular forms of cancer. Although the correlation between cancer and thrombosis is well‐known, it is important to mention that, currently, there has been no research conducted to specifically examine the factors that contribute to VTE in colon cancer patients from China. To the best of our understanding, this research is the initial one to utilize a substantial sample size in creating a nomogram model to precisely forecast the risk of VTE in the overall populace with Stages I–IV colon cancer in China. This research contributes to filling a critical gap in understanding and addressing the risk factors associated with VTE in Chinese diagnosed with colon cancer.

Various studies have reported on the VTE risk in colon cancer patients in other countries. Discrepancies in results among these studies can likely be attributed to differences in population size, study design, and methodologies employed.^11^, ^27^ It is essential to recognize that findings from these studies may not be directly applicable to colon cancer patients in China. The nomogram could simplify the prediction of the outcome event by transforming a complicated regression equation into a visually intuitive graph.^17^ Leveraging the strengths of the nomogram model and considering the specific characteristics of the Chinese population, our study has successfully developed a prediction model for VTE risk in colon cancer patients. Differs from previous studies, our population‐based cohort study in terms of a wider age range, inclusion of various TNM stages, and consideration of multiple clinical factors. Importantly, it includes both surgical and non‐surgical patients, providing ample power for robust analyses.

We can conclude that nomogram performs better than KS and the predictive abilities for VTE of nomogram superior to that of KS in low‐ and high‐risk groups. The results were similar to some previous studies^23^, ^24^ which indicated that KS performed moderately in predicting VTE risk. For instance, in a study concerned about cancer‐associated thrombosis in two unique US health care systems conducted by Li et al.,^16^ they derived and externally validated a new clinical risk assessment model (RAM) for VTE in cancer patients who received modern systemic therapy. Compared with the original KS, the RAM had improved performance that it reclassified 28% of patients and approximately doubled the number of VTE events in the high‐risk group (from 37% to 68%). The DCA curves of KS showed little clinical value. However, the net benefits of nomogram model in the study were higher than those of KS, emphasizing nomogram model's greater potential for guiding personalized treatment decisions.

Our research displayed that elder individuals had a higher probability of being diagnosed with VTE, which is consistent with the results of prior investigations.^10^, ^13^, ^28^ Additionally, the association between a higher VTE incidence and chemotherapy or targeted therapy were similar to the findings of previous studies. Cancer patients who underwent specific cytotoxic chemotherapy regimens had a higher chance, around 2.2 times, of developing VTE compared to those who did not treat with chemotherapy.^10^ Moreover, the research of Ho‐Young Yhim et al.^29^ showed a significant increase in the likelihood of blood clotting events, such as VTE, in individuals with recurrent/metastatic colorectal cancer who received cetuximab plus combination chemotherapy as their first‐line treatment. In our study, 10.8% (125 out of 1156) of colon cancer patients who received chemotherapy experienced VTE. A retrospective study^30^ affirms our findings, reporting a 10.9% annual VTE incidence in colorectal cancer patients treated with chemotherapeutic regimens. These results collectively emphasize the importance of recognizing and addressing the heightened risk of VTE in colon cancer patients undergoing specific treatment modalities, particularly chemotherapy.

In our study, hypertension emerged as an independent risk predictor of VTE in colon cancer patients. This finding aligns with that of Yuhui Zhang et al.'s study^31^ that hypertension was found to be linked with an increased risk of VTE in recently diagnosed lung cancer, regardless of the type and stage of the tumor. High blood pressure as a traditional risk factor for heart diseases, the reason behind its connection with the emergence of VTE probably resides in abnormalities within the blood clotting system, impaired functioning of blood vessel lining, and inflammation of blood vessels.^31^ Moreover, the development of cancer has been associated with a rise in substances that promote blood clotting, like tissue factor, cancer procoagulant, and factor VIIa. This contributes to excessive blood clotting and increases the likelihood of VTE.^13^ In contrast, diabetes was not found to be causally related to the development of VTE in our study. The relationship between diabetes and VTE is controversial. For example, in a significant group of cirrhotic patients who were admitted to the hospital, diabetes was identified as a separate indicator of portal vein thrombosis.^32^ Also, another recent study^33^ reported that diabetes is a possible risk factor for VTE after arthroscopic shoulder surgery. However, a study using bidirectional two‐sample Mendelian randomization^34^ did not find any positive impacts of VTE on both Type 1 and Type 2 diabetes. The authors proposed that in observational research, the notable correlation might be due to confounding factors (like tobacco use, ethnicity, and BMI) commonly found in individuals with diabetes, rather than a direct consequence of this disease. They believe that diabetes is not an independent predictive factor of VTE in the absence of the influence of reverse causality and environmental confounding. Additional investigation is required to clarify these intricate connections.

The correlation between hypercoagulable state and the risk of VTE were evaluated in our research using various screening tests, including PT, APTT, PLT, and D‐dimer levels. Our findings indicated that colon cancer patients who experienced VTE had a longer PT and/or a lower PLT level, and these differences were significant when compared to those without VTE in the multivariable logistic regression. A probable reason is the presence of numerous tissue factors in plasma when PLT levels decline. These tissue factors can convert PLT into thrombin, which in turn aggravate the severity of bleeding caused by the clotting system, leading to a prolonged time required for plasma coagulation. This relationship between PLT levels and VTE differs from the findings of Masataka Ikeda et al.,^11^ who reported a correlation between higher PLT levels and the presence of VTE in univariable analysis. The variation in the study sample could account for this inconsistency. In our study, the majority of patients were in Stage IV, possibly receiving stronger and more frequent doses of chemotherapy drugs, which cumulatively impact PLT levels and contribute to complications such as a drop in PLT levels over time. While the proportion of patients in Stage IV was less than patients in Stages II and III in the Masataka Ikedal et al.'s study.^11^ Furthermore, our results revealed that patients with VTE were associated with lower APTT level. According to the report of Zakai et al.,^35^ a decreased APTT was linked to a twofold rise in the risk of VTE during a 13‐year follow‐up. Another study^36^ that included 5620 patients with lung, gastric, pancreatic cancer, and lymphoma found that APTT had a directly proportional and inversely related connection to the risk of VTE. The existence of hypercoagulability could be the explanation of this phenomenon. Furthermore, our results are consistent with prior evidence, indicating that an elevated D‐dimer concentration is autonomously linked to an elevated likelihood of VTE in individuals diagnosed with colorectal cancer. In the study of Maki Oi et al.,^37^ patients with elevated D‐dimer levels had a greater likelihood of experiencing long‐term recurrent VTE, and there was a higher occurrence of both overall and severe PE. D‐dimer's biological function involves participating in hemostasis and fibrinolytic processes. In the process of thrombosis, plasmin is activated to degrade fibrin into D‐dimer and other fibrin degradation products. Hence, the D‐dimer level can reflect the activity of thrombosis and fibrinolysis processes, serving as a crucial indicator for anticipating occurrences of VTE during the treatment of patients.

Recognizing the constraints of our research is crucial. First, being a single‐center study, there may be inherent biases in patient inclusion, potentially affecting the generalizability of the findings. Second, as a retrospective study, it carries inherent limitations, such as the reliance on historical data and the potential for inconsistent records. Third, the study lacked external validation. Additionally, some studies^38^ have suggested that genetic and epigenetic oncogenic drivers of cancer cells may be associated with the occurrence of cancer‐associated thrombosis. Regrettably, the data regarding driver genes was not available in our study. Therefore, a prospective study with detailed genetic information conducted across multiple centers with external validation to improve the quality of results and make the prediction model more credible is the further direction, allowing for a more comprehensive exploration of the factors influencing cancer‐associated thrombosis.

## 5 CONCLUSIONS

The establishment and internal validation of a novel nomogram for predicting VTE risk in colon cancer patients in China, with a large sample size, wide age range, and consideration of multiple clinical factors, mark a significant contribution to the field. Physicians can utilize this nomogram as a valuable resource to accurately pinpoint patients who are at a heightened risk of VTE. The implementation of this nomogram enables early adoption of preventive and treatment strategies, ultimately reducing the likelihood of thrombogenesis. The practical application of this model in clinical settings holds the potential to enhance patient care and outcomes in the context of colon cancer.

## AUTHOR CONTRIBUTIONS

**Yuanyuan Yang:** Conceptualization (lead); formal analysis (equal); investigation (equal); methodology (equal); writing – original draft (equal). **Jiayi Zhan:** Conceptualization (supporting); investigation (equal); methodology (equal); writing – original draft (equal). **Xiaosheng Li:** Conceptualization (supporting); investigation (equal); methodology (equal); writing – original draft (equal). **Jun Hua:** Data curation (lead); formal analysis (equal). **Haike Lei:** Methodology (equal); project administration (supporting); software (lead); supervision (equal); writing – review and editing (equal). **Xiaoliang Chen:** Project administration (lead); supervision (equal); writing – review and editing (equal).

## FUNDING INFORMATION

No funding was received.

## CONFLICT OF INTEREST STATEMENT

The authors report no conflict of interest.

## ETHICS STATEMENT

The study was conducted according to the Declaration of Helsinki's ethical standards for medical research involving human people. The protocol was reviewed and approved by the Ethics Committee of Chongqing University Cancer Hospital (CZLS2023337‐A). Written consent was waived in the retrospective study.

## Data Availability

The datasets utilized and/or examined in the present investigation can be obtained from the corresponding author upon a reasonable inquiry.
