# Correction to “A nomogram to predict the risk of venous thromboembolism in patients with colon cancer in China”

**DOI:** 10.1002/cam4.7321

**Published:** 2024-05-24

Yang Y, Zhan J, Li X, Hua J, Lei H, Chen X. A nomogram to predict the risk of venous thromboembolism in patients with colon cancer in China. *Cancer Medicine*. 2024;13(9):e7231. doi: 10.1002/cam4.7231.

Figure 3 and Figure 4 were originally in the wrong order. The correct order is shown below:

**FIGURE 3.** Calibration plots of the nomogram for VTE risk in the training cohort (A) and internal validation cohort (B). VTE, venous thromboembolism.

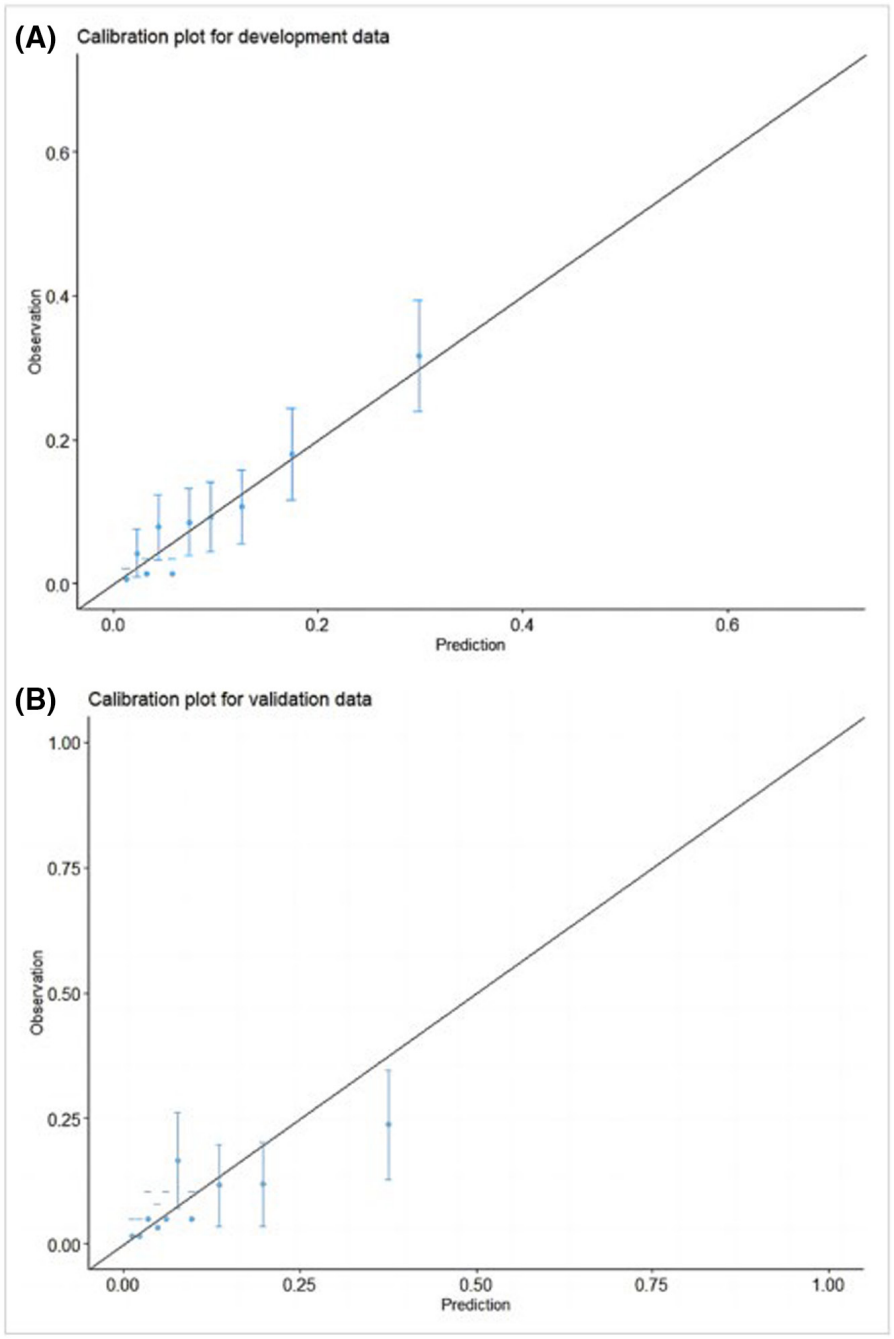

**FIGURE 4.** ROC curves of the nomogram (A) and KS (B) for VTE risk prediction in the training and internal validation cohorts. KS, Khorana score; ROC, receiver operating characteristic curve; VTE, venous thromboembolism.

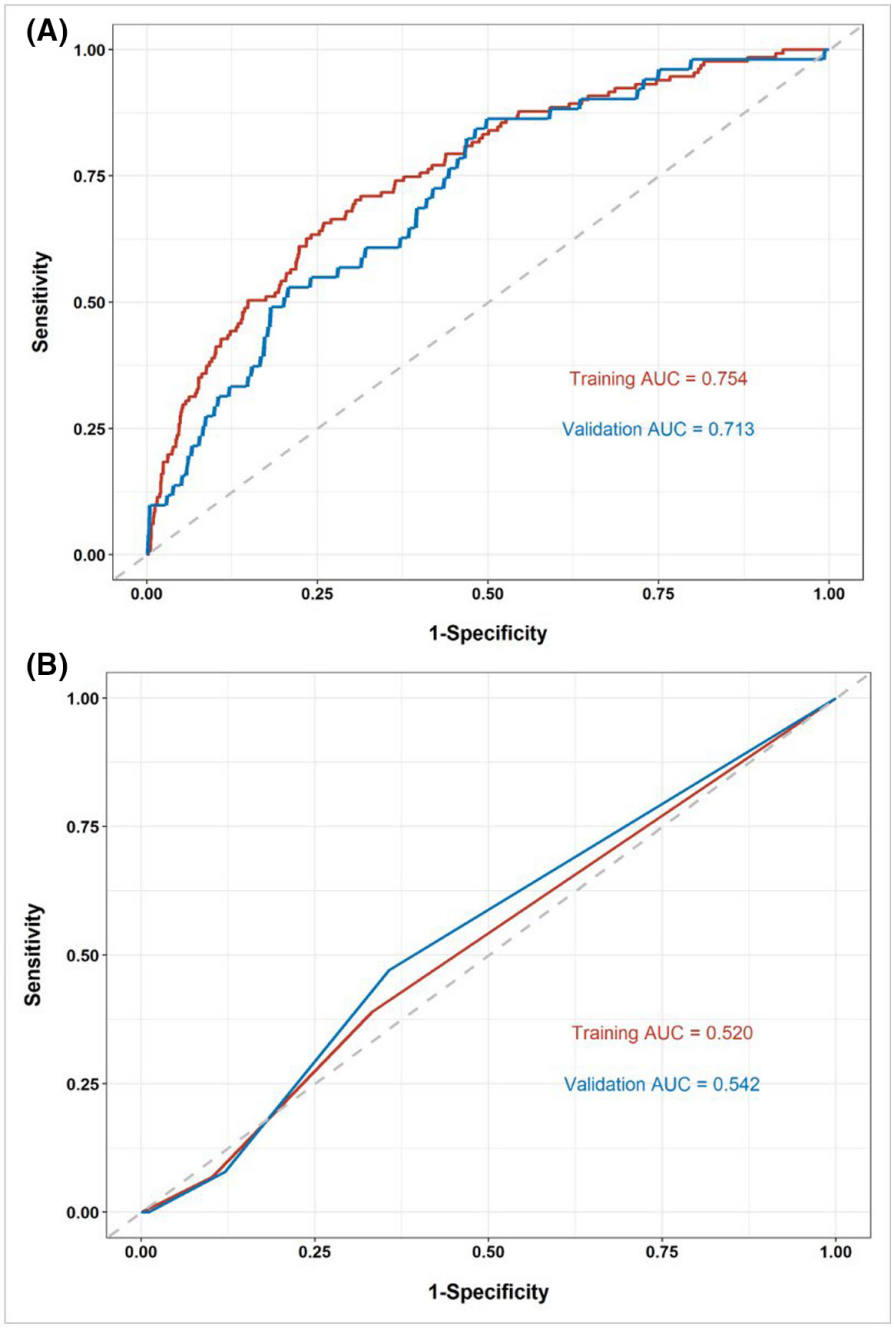

We apologize for this error.

